# Nutritional status, metabolic changes and white blood cells in adolescents☆

**DOI:** 10.1016/j.rpped.2014.04.004

**Published:** 2014-12

**Authors:** Thatianne Moreira Silva Oliveira, Franciane Rocha de Faria, Eliane Rodrigues de Faria, Patrícia Feliciano Pereira, Sylvia C.C. Franceschini, Silvia Eloiza Priore

**Affiliations:** a Universidade Federal de Viçosa (UFV), Viçosa, MG, Brazil; b Universidade Federal do Espírito Santo (UFES), Alegre, ES, Brazil

**Keywords:** Obesity, Adolescent, Risk factors, Leukocyte count

## Abstract

**OBJECTIVE:**

To analyze the relationship between the peripheral blood white cells, metabolic changes, and nutritional status of adolescents with and without excess weight and body fat.

**METHODS:**

This cross-sectional study evaluated the body mass index (BMI) and percentage body fat (%BF) in 362 adolescents from 15 to 19 years of age, of both sexes. White blood cell count, platelet count, uric acid, fasting glucose, insulin, and lipid profile were measured. The inclusion criteria were agreement to participate in the study and signature of the informed consent. Exclusion criteria were: presence of chronic or infectious disease; use of medications that could cause changes in biochemical tests; pregnancy; participation in weight reduction and weight control programs; use of diuretics and laxatives; or the presence of a pacemaker. The following statistical tests were applied: Kolmogorov-Smirnov test, Student's t or Mann-Whitney test, Pearson or Spearman correlation tests, and chi-squared test, considering p<0.05.

**RESULTS:**

Overweight was observed in 20.7% of adolescents. The total cholesterol (TC) had a higher percentage of inadequacy (52.2%), followed by high-density lipoprotein (HDL) (38.4%). There was a positive correlation between white cells and serum lipids, insulin, body fat, and BMI. Monocytes were negatively correlated with BMI, and rods with BMI, body fat, and insulin.

**CONCLUSIONS:**

Nutritional status is related to an inflammatory process, and adolescents with excess weight or body fat presented higher amounts of white blood cells.

## Introduction

Adolescence corresponds to the stage of life between childhood and adulthood, from 10 to 19 years of age, during which physical, psychological, and social changes occur, with focus on growth, with an increase in weight and height and sexual maturation.^1^^,^ 2

This is one of the critical periods for the onset of obesity. Approximately 70% of obese adults started to gain weight during adolescence.^4^ Although obesity is associated with several medical complications in adults, the implications of obesity in children and adolescents are yet to be clearly defined.^3.4^

The prevalence of obesity shows increasingly high numbers. It is estimated that in 2030, there will be a worldwide increase of 25% and 32% in cases of overweight and obesity, respectively.^5^ According to the Pan American Health Organization (PAHO), obesity affects all age ranges.^5^ However, in recent decades, the number of overweight adolescents has increased by approximately 70% in the U.S. and by 240% in Brazil.^5^^,^ 6

Obesity, which should be considered a low-level inflammatory condition, is a pro-inflammatory state with hypertrophy and hyperplasia of adipocytes related to metabolic and cardiovascular disorders, such as type 2 diabetes, hypertension, atherosclerosis, dyslipidemia, and acute and chronic inflammatory processes. This is due to the fact that the white adipose tissue produces cytokines or adipocytokines involved in this process.^7^^-^ 10

White blood cells or leukocytes are immune defense system cells and are closely linked to the thrombogenic and inflammatory profile, and their levels are associated with metabolic and cardiovascular disorders caused by obesity.^11^^,^ 12 The change in concentrations of serum lipids can lead to thrombus formation inside arteries and veins, leading to the aggregation of inflammatory markers such as platelets and leukocytes.^7.13^ The levels of neutrophils and eosinophils, as well as monocytes and lymphocytes in obese children, may be important in understanding the evolution of inflammation and disease.^3^

Therefore, this study aimed to correlate white blood cells to metabolic and nutritional alterations in adolescents with and without excess weight and body fat. 

## Method

This was a cross-sectional study conducted in the city of Viçosa, state of MG, between 2011 and 2012, in adolescents aged 15 to 19 years, of both genders, enrolled in public and private schools in the urban area of the municipality.

The sample of 362 adolescents was calculated using Epi Info software, release 6.04 (Centers for Disease Control and Prevention Georgia, United States), based on a specific formula for cross-sectional studies. This study considered a population of 3,608 adolescents in the study age range, a prevalence of 50%, as the study considered as outcome multiple cardiovascular risk factors, acceptable variability of 5%, and a confidence level of 95%.

Adolescents were chosen by drawing lots. Sample selection occurred in all high schools of the municipality. The principals were contacted and were informed about the objectives and methodology of the project, whereupon permission was requested to invite the adolescents to participate in the study. Parents or guardians of students younger than 18 received the informed consent, as well as students aged 18 years and older, and those interested in participating in the study signed it. 

The screening for the selection of suitable volunteers was performed according to the general inclusion criteria, as follows: age between 15 and 19 years; accepting to take part in the research, signing the informed consent. Exclusion criteria were: presence of chronic or infectious diseases; regular use of medications that could cause changes in biochemical tests; pregnancy; having participated in programs of weight reduction and control; use of diuretics/laxatives; or use of a pacemaker.

Anthropometric assessment was performed by nutritionists from the Department of Health of Universidade Federal de Viçosa (UFV). Weight was obtained using a portable electronic digital scale with a maximum capacity of 150kg. Height was determined using a vertical stadiometer with a maximum height of 2.13m. Measurements were performed in duplicate, using the mean value of two measurements. After the data was obtained, the body mass index (BMI) was calculated and the corresponding percentiles according to age and gender was used to classify the nutritional status of adolescents, as proposed by the World Health Organization (WHO).^14^

To assess the percentage of body fat, a vertical tetrapolar bioelectrical impedance device with eight-point tactile electrodes was employed. The examination was performed in the morning after participants fasted for 12 hours, according to the evaluation protocol.^15^ The percentage of body fat was analyzed according to the classification proposed by Lohman.^16^

A 2-meter long inelastic measuring tape was used to measure waist circumference, obtained at the lowest horizontal circumference, and hip circumference, with both measures obtained in duplicate and using the mean value of the two measurements.

The biochemical assessment was conducted in the Laboratory of Clinical Analysis of the Health Division of UFV. White blood cell (WBC) and platelet counts, uric acid, fasting glucose and insulin levels, and lipid profile (total cholesterol [TC], triglycerides [TG], low density lipoprotein [LDL], high density lipoprotein [HDL], and very low density lipoprotein [VLDL]) were assessed. 

The samples with 12 mL of blood were collected in disposable vials by venipunctture in the morning, after a 12-hour fast. WBC and platelet counts were measured by flow cytometry method and uric acid by the enzymatic colorimetric method in automated Cobas Mira Plus equipment (Roche¨ - Indiana, United States) and classified according to gender and age, using the reference values ​​of the Bioclin-Quibasa kit (Bioclin-Quibasa _ Minas Gerais, Brazil), routinely used in the laboratory, which are 2.0-7.0mg/dL for males and 1.5-6.0mg/dL for females.^17^ The cutoff points used in the classification of dyslipidemia were recommended by I Guideline for the Prevention of Atherosclerosis in Childhood and Adolescence.^18^

Altered fasting glucose were considered when levels were >100mg/dL, according to the recommendation of the American Diabetes Association.^19^ For the analysis of hyperinsulinemia, fasting plasma insulin >

15μU/mL was considered altered.^19^ Insulin resistance was calculated by Homeostasis Model Assessment - Insulin Resistance (HOMA-IR), and was considered indicative of insulin resistance when HOMA-IR 33.16.^19^

The values found for waist circumference, hip circumference, WBCs, platelets, and uric acid were classified using a cutoff of 390th percentile, as cutoff points for adolescents are yet to be established. 

Blood pressure (BP) was measured using an automatic BP monitor, and the cutoff points used were those recommended by the Brazilian Society of Cardiology, according to the guidelines of the VI Brazilian Guidelines on Arterial Hypertension.^20^

The data were entered into an Excel 2007 spreadsheet and the analysis was performed using SPSS(r), release 17.0 (Chicago, United States) and Epi-Info software, release 3.5.1 (Centers for Disease Control and Prevention, Georgia, United States). The Kolmogorov-Smirnov test; Student's t-test or the Mann-Whitney test; Pearson's or Spearman's correlation; and the chi-squared test were used. Significance level was set at p<0.05.

The study was approved by the Ethics Committee on Human Research of UFV (Ref. No. 0140/2010). 

## Results

The study included 362 adolescents with a mean age of 17.3 ± 1.2 years. According to the nutritional status classification, adolescents were divided between the normal weight and the excess weight group; as well as the group with and without excess body fat. Regarding the classification of nutritional status by BMI, females accounted for 55% of the normal weight group and 52% of the excess weight group. As for the percentage of body fat, females represented 29% of the group without excess body fat and 79% of the group with excess body fat. Table 1 shows the anthropometric and biochemical variables in relation to nutritional status defined by BMI. Higher values of all parameters were found in adolescents ​with excess weight, with the exception of HDL, which was lower. Leukocytes, eosinophils, band cells, segmented neutrophils, lymphocytes, and monocytes showed no significant differences between adolescents with normal and excess weight. 

**Table 1:**
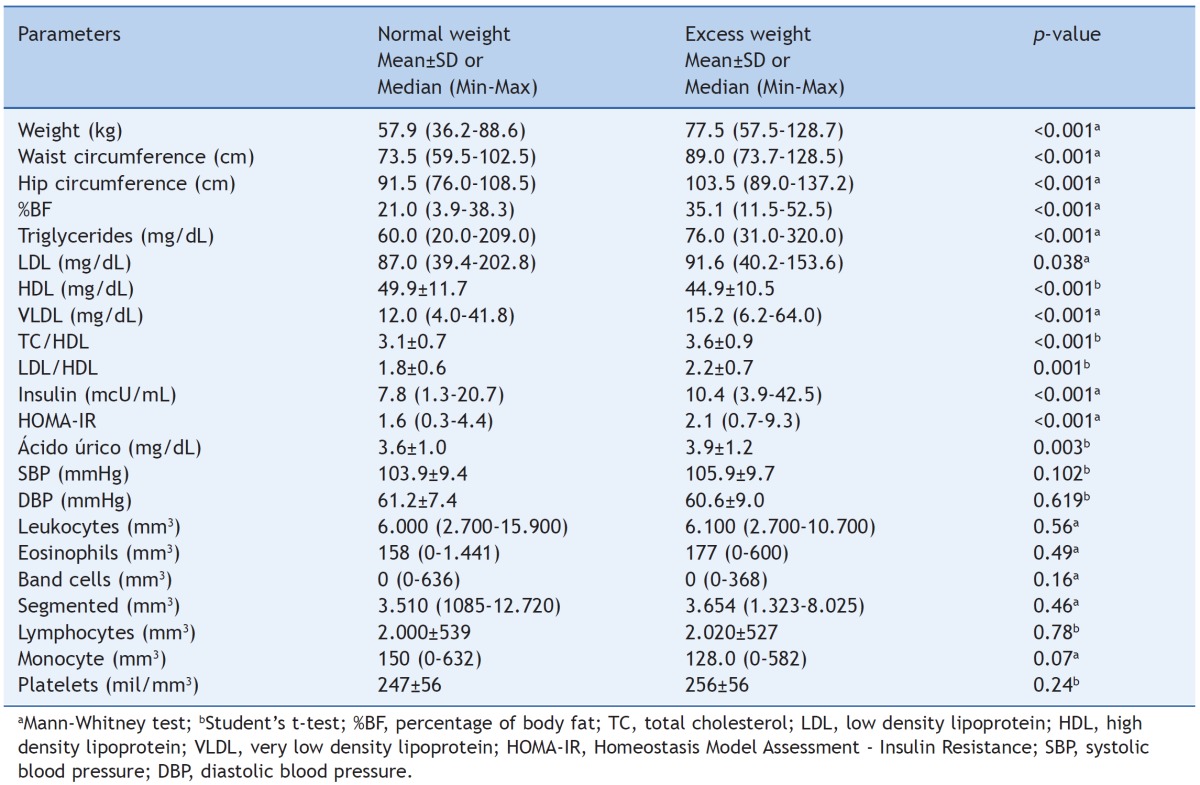
C Comparison of body composition and biochemical variables between groups of adolescents with normal weight and those with excess weight. Viçosa, MG, Brazil.

Table 2 shows the anthropometric and biochemical variables in relation to body fat percentage. Higher values were for all variables ​​in adolescents with excess body fat, with the exception of glucose, uric acid, and systolic blood pressure (SBP), which showed higher values ​​in adolescents without excess body fat. 

**Table 2:**
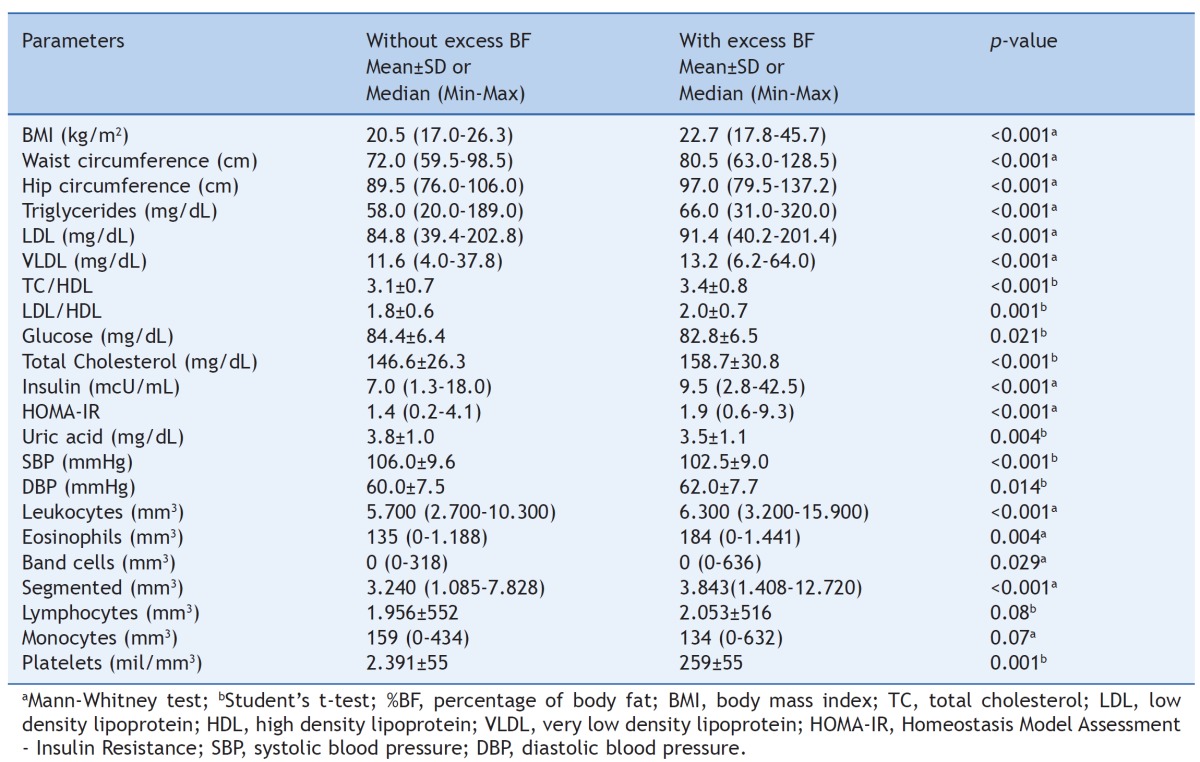
C Comparison of body composition and biochemical variables between groups of adolescents with and without excess body fat. Viçosa, MG, Brazil.

Table 3 shows the anthropometric and biochemical variables in relation to gender. Higher values ​​of weight, glucose, uric acid, and SBP were found in male adolescents, whereas the other variables showed higher values ​​in females. 

**Table 3:**
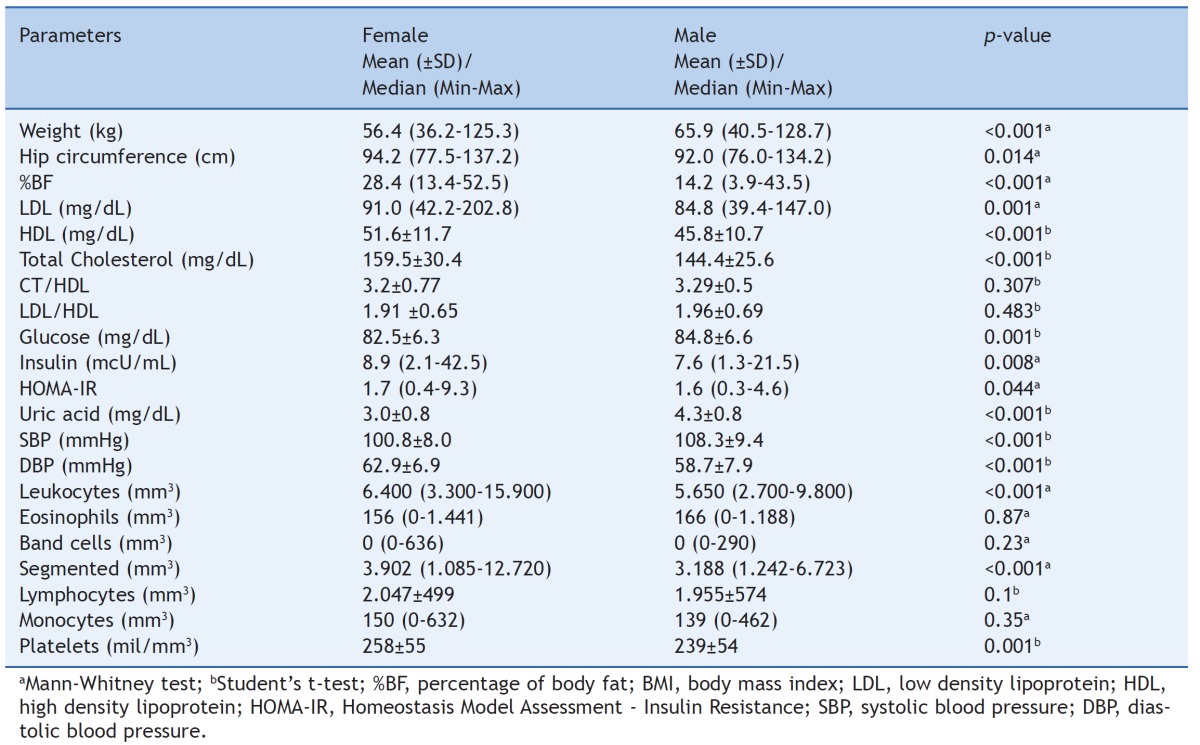
C Comparison of body composition and biochemical variables between genders. Viçosa, MG, Brazil.

Table 4 shows the correlation between white blood cells and platelets with serum lipids, body fat, and insulin, and all variables showed positive correlations with the exception of monocytes, which were negatively correlated with BMI, as well as band cells with BMI, body fat, and insulin. 

**Table 4:**
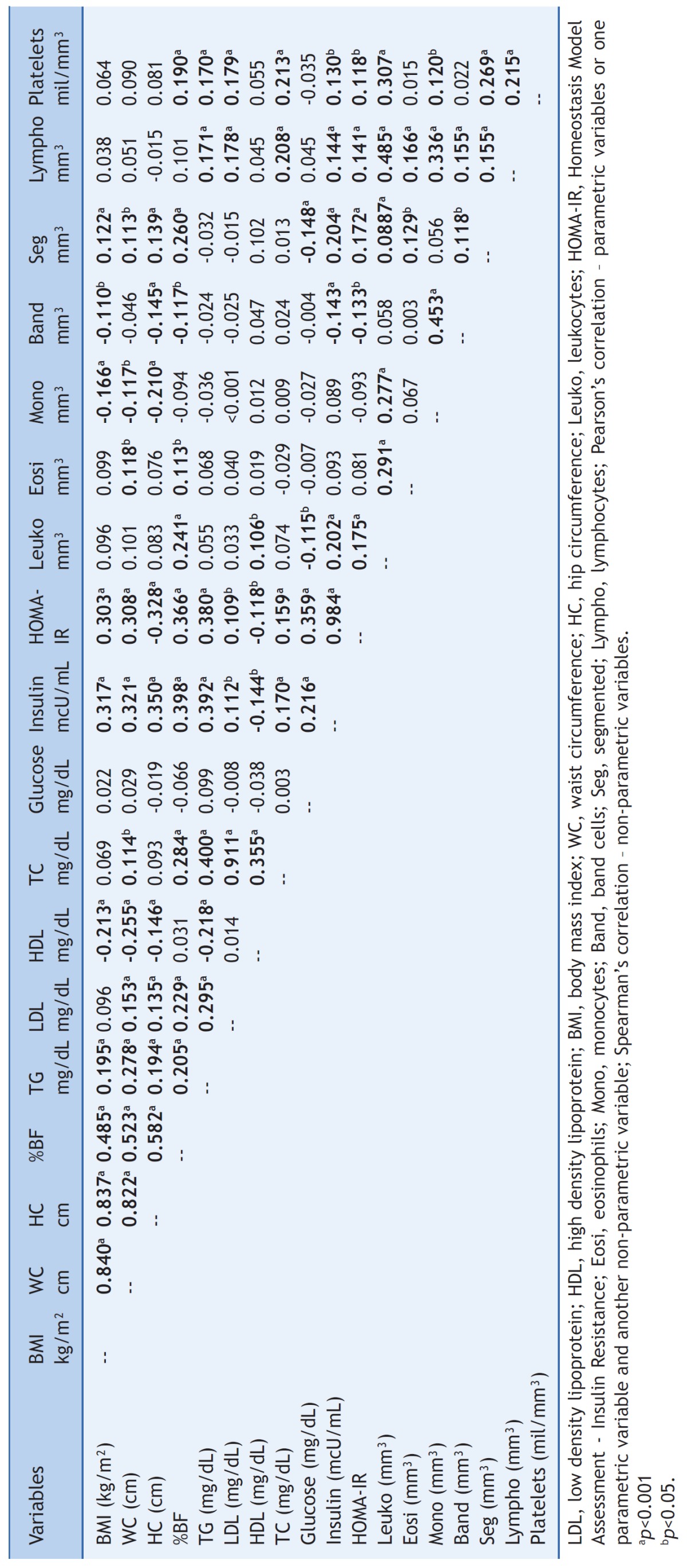
Correlations between body fat, serum lipids, and white blood cells and platelets. Viçosa, MG, Brazil

The nutritional status of the adolescents was associated only with HDL (p<0.001), TG (p=0.004), TC/HDL (p=0.001), and uric acid (p=0.039). The group of adolescents with excess weight was more likely to have low HDL (OR=2.43, 95% CI=1.4-4.23, p<0.001), hypertriglyceridemia (OR=2.44, 95 CI%=1.24-4.79, p=0.004) and hyperuricemia (OR=2.1, 95% CI=0.96-4.56, p=0.039) (Table 5). 

**Table 5:**
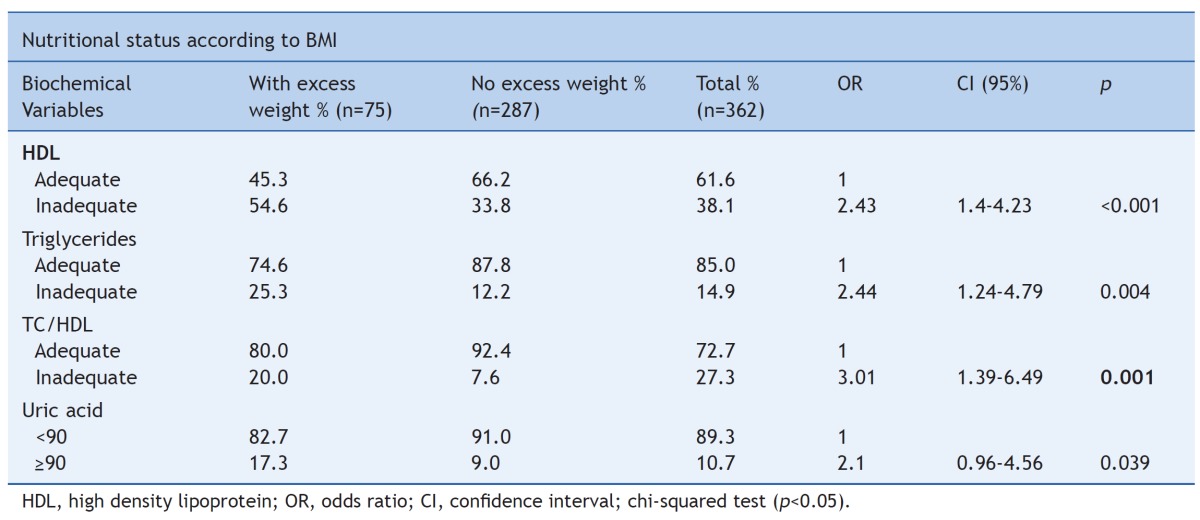
Prevalence of biochemical abnormalities in adolescents with and without excess body weight. Viçosa, MG, Brazil.

Only the lipid profile (TC, p=0.02; TG, p<0.001, LDL p=0.02, and VLDL, p=0.01), insulin (p<0.001), leukocytes (p=0.003), and segmented neutrophils (p=0.02) were associated with excess adiposity; it may be that adolescents with excess weight could have a greater chance of having dyslipidemia, hyperinsulinemia, and a more marked inflammatory state (Table 6). 

**Table 6:**
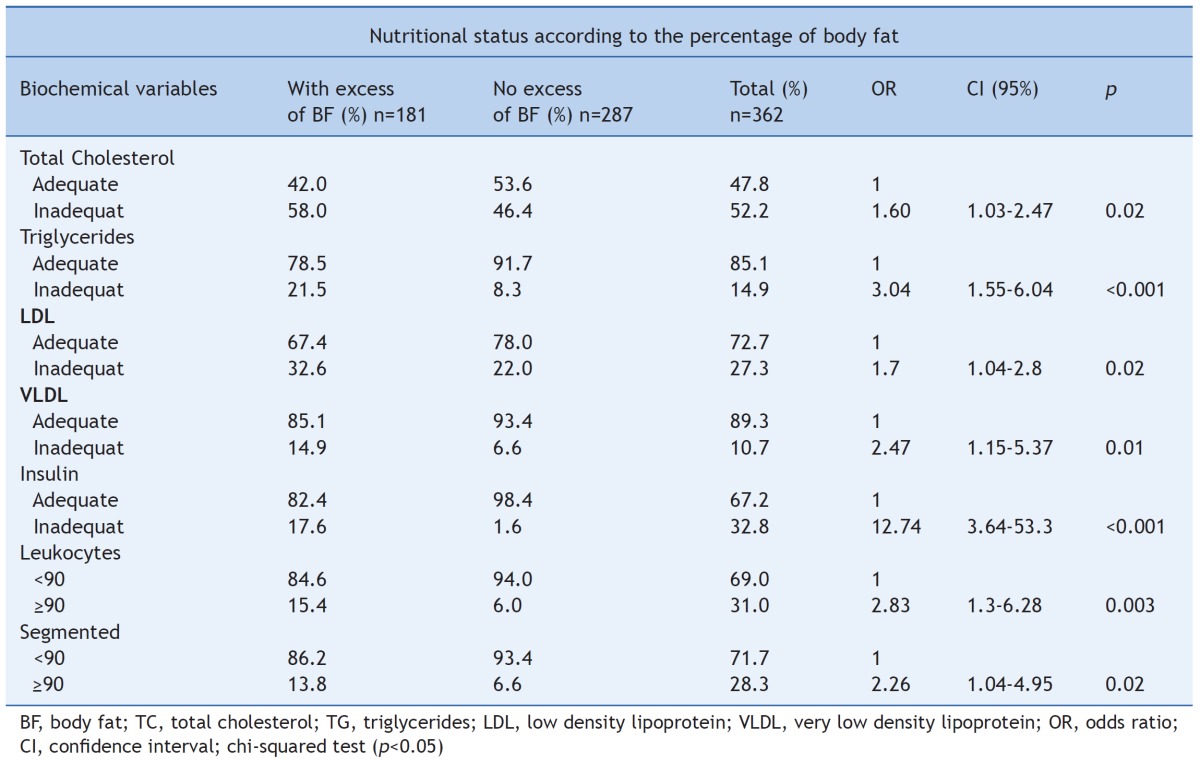
Prevalence of biochemical changes and white blood cells in adolescents with and without excess body fat. Viçosa, MG, Brazil.

The nutritional status of adolescents in relation to gender showed an association only with TC (p<0.001), band cells (p=0.07), segmented neutrophils (p<0.001), and platelets (p=0.03). The female adolescents had a greater chance of having hypercholesterolemia and a more acute inflammatory state, as they had higher levels of cholesterol, neutrophils, and platelets (Table 7). 

**Table 7:**
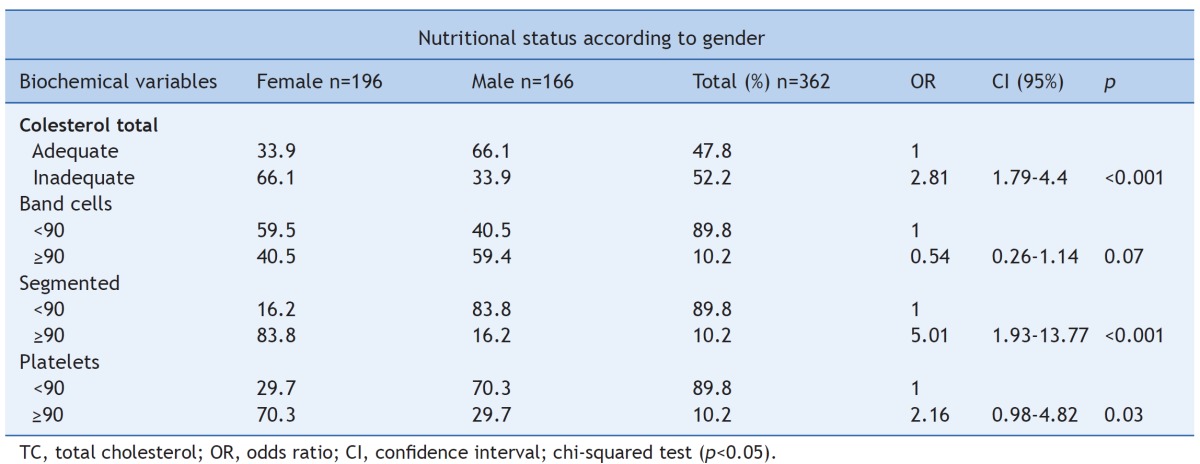
Prevalence of biochemical changes and white blood cells between genders. Viçosa, MG, Brazil.

## Discussion

The 2008-2009 Family Budget Survey (FBS) showed that among males aged 10-19 years of age, the frequency of excess weight increased from 3.7% (1974-75) to 21.7% (2008-09); and in females, the increase of excess weight was 7.6% to 19.4% in the same age group.^21^ The increasing prevalence of overweight and obesity at increasingly younger ages has been a concern of researchers and health care professionals, as excess weight increases the risk of cardiovascular diseases.^5^

In the present study, it was observed that the group with excess weight showed higher prevalence of low HDL levels, hypertriglyceridemia, high TC/HDL ratio, and hyperuricemia. 

The association between dyslipidemia and obesity, previously only seen in adults, has been documented in children and adolescents. According to Priore *et al* 4 overweight students are 2.4 to 7.1-fold moore likely to have higher levels of TC, LDL, and TG; and 12.6-fold more likely to have hyperinsulinemia. However, mean values ​​of HDL were lower among those with excess weight,^4^ results similar to those obtained in the present study.

Currently, it is known that abdominal fat seems to be more associated with dyslipidemia, arterial hypertension, and impaired glucose metabolism, with waist circumference considered a good indicator of adiposity and cardiovascular risk.^10^^,^ 22 Gontijo *et al*,^23^ in their study of 199 adolescents aged 10 to 19 years, observed higher mean values dy of 199 adolescents aged 10 to 19 years, observed higher mean values ​​of VLDL, waist circumference, and hip circumference in adolescents with excess weight, similar results to those obtained in the present study.

The accumulation of abdominal fat and hyperinsulinemia are also associated with an inflammatory profile, which generates atherosclerosis, as well as a thrombogenic profile, in which the number of WBCs appears to be increased.^12^^,^ 24 This may explain the higher number of leukocytes, eosinophils, and segmented neutrophils found in the adolescents with excess body fat in this study. 

To prevent the occurrence of atherosclerosis, an inflammatory picture is initiated, in which WBCs are recruited to the site of the vessel with fat accumulation, in order to prevent thrombus formation.^25^ This inflammatory process can explain the positive correlation between leukocytes and lymphocytes with body fat, TC, LDL, and TG. 

Regarding body weight, there was no difference between concentrations of WBCs (lymphocytes, monocytes, neutrophils, eosinophils, and band cells) between adolescents with and without excess weight. Foschini *et al*,^26^ when assessing 48 adolescents, 27 obese and 21 non-obese according to BMI, also reported no differences regarding the concentrations of leukocytes, neutrophils, lymphocytes, and monocytes. However, Zaldivar *et al* 3 have shown that obese children have a higher concentration of circulating leukocytes, particularly neutrophils, monocytes, and lymphocytes. Although the mechanisms of these increases are not well understood, it is known that child and adult obesity is associated with increased levels of circulating cytokines such as interleukin-6 (IL-6) and tumor necrosis factor alpha (TNF-±), and may contribute to an elevation in the number of circulating leukocytes.^26^

Moreover, Foschini *et al*.^26^ found a higher concentration of platelets in obese adolescents, as well as higher levels of platelets in adolescents with excess body fatt. 

Platelet activation and aggregation are the main processes in the pathophysiology of cardiovascular disease. Mean platelet volume (MPV), responsible for platelet activation, emerges as a new risk marker for atherothrombosis.^26^

In a study of 38 boys and girls, aged 6 to 18 years old, the authors found an overall increase in total leukocyte count (*p*=0.011) in the group with excess weight. Increases in the number of monocytes (*p*=0.008) were also observed in the same group. In the present study, the increase in the number of leukocytes was demonstrated in the group of adolescents with excess body fat and a negative correlation of monocytes with BMI was observed (r=-0.166, *p*=0.001). As for the number of eosinophils and lymphocytes, there was no difference (*p*>0.05) between groups with and without excess weight in the study of Zaldivar *et al*,^3^ as well as in the present study. 

The increase in the number of leukocytes observed in adolescents with excess body fat is similar to the results that have been reported in adults. A high leukocyte count was found to be an independent risk factor for coronary heart disease, so that a reduction of 1 billion in the total leukocyte count may result in a 14% decrease in the risk of coronary heart disease.^3^

Bao *et al* 27 suggested that girls may have higher overall counts than boys. According to the study, women showed higher number of leukocytes and higher prevalence of elevated levels of segmented neutrophils. 

Zaldivar *et al* 3 also found a strong association between body fat and leukocytes and a reasonable correlation between BMI and WBCs, and these results were also found in the present study. 

The accumulation of fat in the abdominal region since adolescence is associated with hyperinsulinemia and elevated levels of certain inflammatory markers, such as IL-6, TNF-± and C-reactive protein (CRP) and WBCs, which are also associated with abdominal obesity. Ganguli *et al*,^28^ in their study of Asian women, found a significant correlation of leukocytes with CRP and a strong associatiion between CRP levels and measures of adiposity, such as BMI, waist circumference, and body fat. There was also evidence of a positive correlation of CRP with components of metabolic syndrome, insulin, and HOMA-IR.^29^ This may explain the results found in this study, regarding the strong correlation between WBCs and body fat, insulin, and HOMA-IR. 

Based on the findings of this study, it was concluded that nutritional status is associated with an inflammatory condition and that adolescents with excess weight and/or body fat had a higher number of circulating WBCs.
