# Supplementary material for: Nutritional status, metabolic changes and white blood cells in adolescents
Source: Rev Paul Pediatr. 2014 Dec;32(4):351–9. doi: 10.1016/j.rpped.2014.04.004 (PMC4311789; doi:10.1016/j.rpped.2014.04.004)
Supplement: Translation

# Translation: Estado nutricional, alterações metabólicas e células brancas na adolescência☆

**Authors:** Thatianne Moreira Silva Oliveira, Franciane Rocha de Faria, Eliane Rodrigues de Faria, Patrícia Feliciano Pereira, Sylvia C.C. Franceschini, Silvia Eloiza Priore

**Affiliations:** a Universidade Federal de Viçosa (UFV), Viçosa, MG, Brasil; b Universidade Federal do Espírito Santo (UFES), Alegre, ES, Brasil

**Keywords:** Obesidade, Adolescente, Fatores de risco, Contagem de leucócitos

## Abstract

**OBJETIVO:**

Analisar a relação entre as células brancas do sangue periférico e as alterações metabólicas e estado nutricional de adolescentes com e sem excesso de peso e gordura corporal.

**MÉTODOS:**

Avaliou-se, em estudo transversal, o Índice de Massa Corporal (IMC) e o percentual de gordura corporal (%GC) em 362 adolescentes de 15 a 19 anos de idade, de ambos os sexos. Os critérios gerais de inclusão foram: ter aceitado participar da pesquisa e assinado o termo de consentimento livre e esclarecido. Os critérios de exclusão foram: relatar a presença de doenças crônicas ou infecciosas; usar medicamentos que pudessem causar alteração nos exames bioquímicos; ter engravidado; ter participado de programas de redução e controle de peso; usar diuréticos/laxantes ou usar marcapasso. Realizou-se leucograma, contagem de plaquetas, ácido úrico, glicemia de jejum, insulina e perfil lipídico. Utilizaram-se os testes Kolmogorov-Smirnov, t de Student ou Mann Whitney, correlação de Pearson ou de Spearman e qui-quadrado, considerando significante *p<*0,05.

**RESULTADOS:**

Excesso de peso foi verificado em 20,7% dos adolescentes. O colesterol total

**(CT):**

apresentou maior porcentagem de inadequação (52,2%), seguido da lipoproteína de alta densidade (HDL) (38,4%). Encontraram-se correlações positivas entre células brancas e lipídeos séricos, insulina, gordura corporal e IMC. Os monócitos apresentaram correlação negativa com IMC e os bastonetes com IMC, gordura corporal e insulina.

**CONCLUSÕES:**

O estado nutricional está relacionado com um quadro inflamatório, sendo que adolescentes com excesso de peso e/ou de gordura corporal apresentaram maiores quantidades de células brancas.

## Introdução

A adolescência corresponde à fase da vida entre a infância e a idade adulta, dos 10 aos 19 anos de idade, período em que ocorrem transformações físicas, psicológicas e sociais, apresentando como foco o crescimento, com o aumento do peso e da estatura e a maturação sexual.^1^^,^ 2

Esta fase é um dos períodos críticos para o aparecimento da obesidade. Aproximadamente 70% dos adultos obesos começaram a ganhar peso no período da adolescência.^4^ Embora, a obesidade esteja associada a numerosas complicações médicas em adultos, as implicações da obesidade na criança e no adolescente ainda não estão claramente definidas.^3^^,^ 4.

A prevalência da obesidade apresenta números cada vez mais altos. Estima-se que, para 2030, haja aumento de 25 e 32% nos casos de sobrepeso e obesidade, respectivamente, em todo o mundo.^5^ Segundo a Organização Pan-americana de Saúde (OPAS), a obesidade atinge todas as faixas de idade.^5^ Entretanto, nas últimas décadas o número de adolescentes obesos aumentou cerca de 70% nos Estados Unidos e 240% no Brasil.^5^^,^ 6

 A obesidade, que deve ser considerada como uma condição de baixo nível inflamatório, é um estado pró-inflamatório com hipertrofia e hiperplasia de adipócitos relacionados a alterações metabólicas e cardiovasculares como diabetes melito tipo 2, hipertensão arterial, arterosclerose, dislipidemias, processos inflamatórios agudos e crônicos. Isso se deve ao fato do tecido adiposo branco produzir citocinas ou adipocitocinas envolvidas nesse processo.^7^^-^ 10

As células brancas ou leucócitos são células de defesa do sistema imunológico e estão intimamente ligadas ao perfil trombogênico e inflamatório, sendo a contagem das mesmas relacionadas às alterações metabólicas e cardiovasculares causadas pela obesidade.^11^^,^ 12 A alteração das concentrações dos lipídios séricos pode levar a formação de trombos no interior de artérias e veias, levando a agregação de marcadores inflamatórios como as plaquetas e leucócitos.^7^^,^ 13 Os níveis de neutrófilos e eosinófilos, bem como monócitos e linfócitos, em crianças obesas, podem ser importantes na compreensão da evolução da inflamação e doenças.

Portanto, o objetivo deste estudo é correlacionar células brancas do sangue às alterações metabólicas e ao estado nutricional, em adolescentes com e sem excesso de peso e de gordura corporal.

## Método

O estudo é do tipo transversal realizado na cidade de Viçosa-MG, entre 2011 e 2012, com adolescentes de 15 a 19 anos de idade, de ambos os sexos, matriculados em escolas públicas e particulares da zona urbana do município. 

A amostra com 362 adolescentes foi calculada por meio do *software Epi Info *versão 6.04, a partir de fórmula específica para estudos transversais. Considerou-se a população de 3608 adolescentes na faixa etária de estudo, prevalência de 50%, visto o estudo considerar como desfecho múltiplos fatores de risco cardiovascular, variabilidade aceitável de 5% e nível de confiança de 95%.

Os adolescentes foram escolhidos por meio de sorteio. A seleção da amostra ocorreu em todas as escolas de ensino médio do município. Os diretores foram contatados e tiveram esclarecimento quanto aos objetivos e metodologia do projeto, sendo solicitada autorização para convidar os adolescentes. Foram entregues os Termos de Consentimento Livre e Esclarecido para que os responsáveis pelos alunos menores de 18 anos interessados em participar assinassem ou os próprios assinassem, no caso dos maiores de idade, e pudessem participar do estudo.

A triagem para seleção de voluntários aptos foi feita conforme os critérios gerais de inclusão, sendo eles: ter entre 15 e 19 anos; ter aceitado participar da pesquisa, ter assinado o termo de consentimento livre e esclarecido. Como critérios de exclusão foram considerados: relatar a presença de doenças crônicas ou infecciosas; usar regularmente medicamentos que pudessem causar alteração nos exames bioquímicos; ter engravidado; ter participado de programas de redução e de controle de peso; usar diuréticos/laxantes ou usar marcapasso.

A avaliação antropométrica foi realizada por nutricionistas na Divisão de Saúde da Universidade Federal de Viçosa (UFV). O peso foi obtido por balança portátil digital eletrônica com capacidade máxima de 150 kg. A estatura foi determinada utilizando-se antropômetro vertical, com extensão de 2,13. A leitura foi feita em duplicata, utilizando-se o valor médio dos dois valores obtidos na medição. Com os dados obtidos, calculou-se o índice de massa corporal (IMC), e através desse, observou-se os percentis correspondentes, de acordo com a idade e o sexo, para classificação do estado nutricional dos adolescentes, segundo proposta da *World Health Organization* (WHO).^14^

Para avaliar o percentual de gordura corporal, utilizou-se bioimpedância elétrica tetrapolar vertical com oito eletrodos táteis. O exame foi realizado no período da manhã, com os participantes em jejum de 12 horas e de acordo com o protocolo de avaliação.^15^ O percentual de gordura corporal foi analisado segundo a classificação proposta por Lohman.^16^

Utilizou-se uma fita métrica inelástica com extensão de 2 metros para aferir o perímetro da cintura, obtido na menor circunferência horizontal, e o perímetro do quadril, sendo ambas as medidas aferidas em duplicata, utilizando-se a média entre os dois valores.

Para a avaliação bioquímica, realizaram-se, no Laboratório de Análises Clínicas da Divisão de Saúde da UFV, os exames de leucograma e contagem de plaquetas, ácido úrico, glicemia de jejum, insulina e perfil lipídico (colesterol total - CT; triglicerídeos - TG; LDL; HDL e VLDL). As amostras, com 12mL de volume de sangue, foram colhidas com material descartável, por punção venosa, no período da manhã, estando os adolescentes em jejum de 12 horas. O leucograma com contagem de plaquetas foi dosado pelo método citometria de fluxo e o ácido úrico pelo método colorimétrico enzimático, com automação pelo equipamento *Cobas Mira Plus *(*Roche Corp*.) e classificado de acordo com o sexo e idade, conforme os valores de referência do *kit Bioclin-Quibasa*, utilizado no laboratório, sendo para o sexo masculino 2,0-7,0mg/dL e, para o feminino, 1,5-6,0mg/dL^17^. Os pontos de corte utilizados na classificação de dislipidemias foram os preconizados pela I Diretriz de Prevenção da Aterosclerose na Infância e na Adolescência.^18^

Considerou-se glicemia de jejum alterada valores >100mg/dL, segundo a recomendação da *American Diabetes Association.* 19 Para análise da hiperinsulinemia, foi considerada insulina plasmática alterada de jejum >15µU/mL.^19^ A resistência insulínica foi calculada por meio do HOMA-IR (*Homeostasis Model Assessment - Insulin Resistance*), sendo considerado presença de resistência insulínica valores de HOMA-IR≥3,16.^19^

Os valores encontrados pelo perímetro da cintura, e perímetro do quadril, células brancas, plaqueta e ácido úrico, foram classificados utilizando o ponto de corte maior ou igual ao percentil 90, uma vez que ainda não existem pontos de cortes definidos para adolescentes. 

A pressão arterial foi aferida utilizando aparelho de pressão sanguínea automático, e os pontos de corte utilizados foram os preconizado pela Sociedade Brasileira de Cardiologia, seguindo as orientações das VI Diretrizes Brasileiras de Hipertensão Arterial.^20^

Os dados foram digitados no software *Excel2007* e as análises realizadas no programa *Statistical Package for the Social Sciences* ^(r)^ *(*SPSS, versão 17.0*) e *no software *Epi-Info* ^(r)^, versão 3.5.1*.* Utilizou-se os testes Kolmogorov-Smirnov; teste *t* de Student ou Mann Whitney; correlação de *Pearson* ou de *Spearman* e qui-quadrado. O valor de significância considerado foi de *p*<0,05.

O estudo foi aprovado pelo Comitê de Ética em Pesquisa com Seres Humanos da UFV (Of. Ref. N° 0140/2010).

## Resultados

Participaram do estudo 362 adolescentes, com idade média de 17,3±1,2 anos. De acordo com a classificação do estado nutricional, os adolescentes foram distribuídos entre o grupo dos eutróficos e dos com excesso de peso e o grupo sem e com excesso de gordura corporal. Com relação à classificação do estado nutricional pelo IMC, o sexo feminino representou 55% do grupo dos eutróficos e 52% do grupo com excesso de peso. Já em consideração ao percentual de gordura corporal, o sexo feminino representou 29% do grupo sem excesso de gordura corporal e 79% do grupo com excesso de gordura corporal. A tabela 1 apresenta os valores das variáveis antropométricas e bioquímicas, em relação ao estado nutricional pelo IMC. Para todos os parâmetros, foram encontrados maiores valores nos adolescentes com excesso de peso, com exceção do HDL que apresentou menor valor. Leucócitos, eosinófilos, bastonetes, segmentados, linfócitos e monócitos não apresentaram diferença significativa entre os adolescentes eutróficos e com excesso de peso. 

**Tabela 1:** Comparação da composição corporal e variáveis bioquímicas entre os grupos dos adolescentes eutróficos e com excesso de peso. Viçosa, MG, Brasil

A tabela 2 apresenta os valores das variáveis antropométricas e bioquímicas, em relação ao percentual de gordura corporal. Para todas as variáveis, foram encontrados maiores valores nos adolescentes com excesso de gordura corporal, com exceção da glicose, do ácido úrico e da pressão arterial sistólica (PAS), os quais apresentaram maiores valores nos adolescentes sem excesso de gordura corporal.

**Tabela 2:** Comparação da composição corporal e variáveis bioquímicas entre os grupos dos adolescentes sem e com excesso de gordura corporal. Viçosa, MG, Brasil

A tabela 3 apresenta os valores das variáveis antropométricas e bioquímicas, em relação ao sexo. Foram encontrados maiores valores de peso, glicose, ácido úrico e PAS em adolescentes do sexo masculino e as outras variáveis mostraram valores maiores no sexo feminino. 

**Tabela 3:** Comparação da composição corporal e variáveis bioquímicas entre os sexos. Viçosa, MG, Brasil

A tabela 4 apresenta a correlação entre as células brancas do sangue e plaquetas com os lipídeos séricos, gordura corporal e insulina, sendo que todas as variáveis apresentaram correlações positivas, com exceção dos monócitos que apresentaram correlação negativa com o IMC, assim como os bastonetes com o IMC, com a gordura corporal e com a insulina.

**Tabela 4:** Correlações entre gordura corporal, lipídeos séricos e as células brancas e plaquetas. Viçosa, MG, Brasil

O estado nutricional dos adolescentes apresentou associação apenas com HDL (*p<*0,001), triglicerídeos (*p=*0,004), CT/HDL (*p=*0,001) e ácido úrico (*p=*0,039). O grupo dos com excesso de peso teve maior chance de apresentar baixos níveis de HDL (OR=2,43; IC 95%=1,4-4,23; *p<*0,001), hipertrigliceridemia (OR=2,44; IC 95%=1,24-4,79; *p=*0,004) e hiperuricemia (OR=2,1; IC 95%=0,96-4,56; *p=*0,039) (tabela 5).

**Tabela 5:** Prevalência de alterações bioquímicas em adolescentes com e sem excesso de peso corporal. Viçosa, MG, Brasil

Apenas o perfil lipídico (CT, *p=*0,02; TG, *p<*0,001; LDL, *p=*0,02 e VLDL, *p=*0,01), a insulina (*p<*0,001), os leucócitos (*p=*0,003) e os segmentados (*p=*0,02) apresentaram associação com o excesso de adiposidade; podendo os adolescentes com excesso de peso ter maior chance de apresentarem dislipidemias, hiperinsulinemia e um quadro de inflamação mais acentuado (tabela 6).

**Tabela 6:** Prevalência de alterações bioquímicas e células brancas entre adolescentes com e sem excesso de gordura corporal. Viçosa, MG, Brasil

O estado nutricional dos adolescentes, com relação ao sexo, apresentou associação apenas com colesterol total (*p<*0,001), bastonetes (*p=*0,07), segmentados (*p<*0,001) e plaquetas (*p=*0,03). O sexo feminino teve maior chance de ter hipercolesterolemia e um quadro mais agudo de inflamação, uma vez que apresentou maiores quantidades de colesterol, segmentados e plaquetas (tabela 7).

**Tabela 7:** Prevalência de alterações bioquímicas e células brancas entre os sexos. Viçosa, MG, Brasil

## Discussão

A Pesquisa de Orçamentos Familiares 2008-2009 (POF) mostrou que, entre os indivíduos do sexo masculino de 10 a 19 anos de idade, a frequência do excesso de peso passou de 3,7% (1974-75) para 21,7% (2008-09); e no feminino, o aumento do excesso de peso foi de 7,6% para 19,4% na mesma faixa etária.^21^ O aumento da prevalência de sobrepeso e obesidade em idades cada vez mais precoces tem preocupado pesquisadores e profissionais da área de saúde, visto que o excesso de peso predispõe ao risco de doenças cardiovasculares.^5^

No presente estudo, observou-se que o grupo com excesso de peso apresentou maiores prevalências para uma baixa quantidade de HDL, hipertrigliceridemia, alta relação de CT/HDL e hiperuricemia.

A associação entre dislipidemia e obesidade, antes somente observada em adultos, vem sendo documentada em crianças e adolescentes. Segundo Priore *et al*,^4^ escolares com sobrepeso apresentam probabilidade de 2,4 a 7,1 vezes maior de ter colesterol total, lipoproteína de baixa densidade (LDL) e triglicerídeos elevados, e 12,6 vezes maior probabilidade de ter hiperinsulinemia. Por sua vez, valores médios de HDL foram inferiores entre os com excesso de peso,^4^ resultados semelhantes aos obtidos no presente estudo. 

Atualmente, sabe-se que a localização abdominal de gordura mostra-se mais associada às dislipidemias, hipertensão arterial e alterações no metabolismo glicêmico, sendo o perímetro da cintura considerado bom indicador da adiposidade corporal e do risco cardiovascular.^10^^,^ 22

Gontijo *et al*,^23^ em seu estudo com 199 adolescentes de 10 a 19 anos, observou valores médios superiores de VLDL, do perímetro da cintura e do quadril entre adolescentes com excesso de peso, resultados semelhantes obtidos no presente estudo. 

O acúmulo de gordura na região abdominal e a hiperinsulinemia estão também associados a um perfil inflamatório, o qual gera aterosclerose, e tombogênico, no qual a quantidade de células brancas aparece aumentada.^12^^,^ 24 Isso pode explicar a maior quantidade de leucócitos, eosinófilos e segmentados nos adolescentes com excesso de gordura corporal, encontrados nesse estudo.

Para evitar a ocorrência da aterosclerose, inicia-se um quadro inflamatório no qual as células brancas são recrutadas para o local do vaso onde se encontra o acúmulo de gordura, com o intuito de evitar a formação de trombos.^25^ Esse processo inflamatório pode explicar a correlação positiva existente entre leucócitos e linfócitos com a gordura corporal, o colesterol total, o LDL, os triglicerídeos. 

Com relação ao peso corporal, não houve diferença entre concentrações de células brancas (linfócitos, monócitos, segmentados, eosinófilos e bastonetes) entre os com e sem excesso de peso. Foschini *et al*,^26^ ao avaliarem 48 adolescentes, sendo 27 obesos e 21 não obesos segundo o IMC, também não relataram diferença com relação às concentrações de leucócitos, neutrófilos, linfócitos e monócitos. Por outro lado Zaldivar *et al*,^3^ demonstraram que crianças obesas apresentam maior concentração de leucócitos circulantes, em particular, neutrófilos, monócitos e linfócitos. Embora os mecanismos dessas elevações ainda não sejam bem entendidos, sabe-se que a obesidade infantil e adulta, está relacionada ao aumento dos níveis de citocinas circulantes, como a interleucina 6 (IL-6) e o fator de necrose tumoral (TNF-α), podendo contribuir para uma elevação do número de leucócitos circulantes.^26^

Além disso, Foschini *et al* 26 encontraram maior concentração de plaquetas nos adolescentes obesos, bem como maiores níveis de plaquetas nos adolescentes com excesso de gordura corporal.

A ativação e agregação de plaquetas são os principais processos na fisiopatologia da doença cardiovascular. O volume plaquetário médio (VPM), responsável pela ativação plaquetária, surge como um novo marcador de risco para aterotrombose.^26^

Em estudo com 38 meninos e meninas, de 6 a 18 anos, foi encontrado aumento global da contagem total de leucócitos (*p=*0,011) no grupo com excesso de peso. Aumentos no número celular de monócitos (*p=*0,008) também foram notados no mesmo grupo. Já no presente estudo, o aumento de leucócitos foi apresentado no grupo dos adolescentes com excesso de gordura corporal e verificou-se correlação negativa de monócitos com o IMC (r=-0,166; *p=*0,001). Já as quantidades de eosinófilos e linfócitos não foram diferentes (*p*>0,05) entre os grupos com e sem excesso de peso, no estudo de Zaldivar *et al*,^3^ assim como no presente estudo.

O aumento de leucócitos observado nos adolescentes com excesso de gordura corporal é semelhante aos resultados que têm sido relatados em adultos. A contagem de leucócitos alta mostrou ser um fator de risco independente para doenças coronarianas, de tal forma que uma redução de 1 bilhão em contagem total de leucócitos pode resultar numa diminuição de 14% no risco de doenças coronarianas.^3^

Bao *et al*,^27^ sugeriram que as meninas podem ter maiores contagens totais do que os meninos. De acordo com o estudo, o sexo feminino apresentou maiores quantidades de leucócitos e maior prevalência nas quantidades elevadas dos segmentados.

Zaldivar *et al*,^3^ também achou em seu estudo uma forte relação entre a gordura corporal e os leucócitos e razoável correlação entre o IMC e as células brancas sanguíneas, sendo esses resultados também encontrados no presente estudo. 

O acúmulo de gordura na região abdominal, desde a adolescência, está associado com a hiperinsulinemia e níveis elevados de certos marcadores inflamatórios como IL-6, TNF- α e a proteína C reativa (PCR) e células brancas que também estão associados à obesidade abdominal. Ganguli* et al*,^28^ em seu estudo com mulheres asiáticas, verificaram uma correlação significativa de leucócitos com PCR e uma forte associação entre os níveis de PCR e medidas de adiposidade, como IMC, circunferência da cintura e gordura corporal. Houve ainda evidências da correlação positiva do PCR com os componentes da síndrome metabólica, a insulina e o HOMA-IR.^29^ Isso pode explicar os resultados achados nesse estudo, para a forte correlação entre as células brancas e gordura corporal, insulina e HOMA-IR. 

A partir dos achados deste estudo, conclui-se que o estado nutricional está relacionado com um quadro inflamatório, sendo que adolescentes com excesso de peso e/ou de gordura corporal apresentaram maiores quantidades de células brancas.
